# 3-(3-Nitro­phen­yl)-1-[4-(prop-2-yn­yloxy)phen­yl]prop-2-en-1-one

**DOI:** 10.1107/S2414314622009579

**Published:** 2022-10-04

**Authors:** Vinaya, Yeriyur B. Basavaraju, Holalagudu A. Nagma Banu, Balakrishna Kalluraya, Hemmige S. Yathirajan, Rishik Balerao, Ray J. Butcher

**Affiliations:** a Department of Studies in Chemistry, University of Mysore, Manasagangotri, Mysore-570 006, India; b Department of Studies in Chemistry, Mangalore University, Mangalagangotri, Mangalore-574 199, India; c Thomas Jefferson High School for Science and Technology, 6560 Braddock Rd, Alexandria VA 22312, USA; d Department of Chemistry, Howard University, 525 College Street NW, Washington DC 20059, USA; Goethe-Universität Frankfurt, Germany

**Keywords:** crystal structure, nitro­benzene, alkyne, chalcone, phenyl ring

## Abstract

The structure of the title compound, C_18_H_13_NO_4_, shows that the whole mol­ecule is almost planar but with a dihedral angle between the two phenyl rings of 19.22 (5)°. The mol­ecules are linked by C—H⋯O inter­actions, forming sheets in the (21  ) plane.

## Abstract 2

The mol­ecule of the title compound is almost planar but with a dihedral angle between the two phenyl rings of 19.22 (5)°. In the crystal, mol­ecules are linked by C—H⋯O inter­actions, forming sheets in the (21 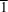 ) plane.

## Graphical abstract

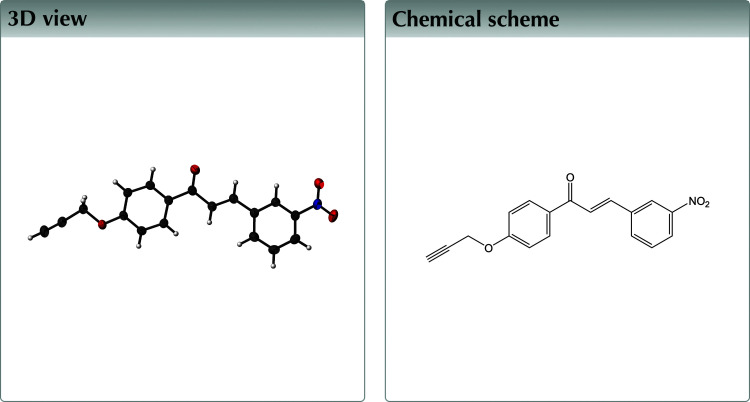

## Structure description

Chalcones are among the leading bioactive flavonoids, with a therapeutic potential implicated to an array of bioactivities that have been investigated by a series of pre­clinical and clinical studies. They contain an α-β unsaturated carbonyl system, which is present in open-chain form, and two aromatic rings are joined through three-carbon atoms (Kozlowski *et al.*, 2007 ▸; Raghav & Garg, 2014 ▸). Studies depicting the biological activities of chalcones and their derivatives describe their immense significance as anti­diabetic, anti­cancer, anti-inflammatory, anti­microbial, anti­oxidant, anti­parasitic, psychoactive and neuroprotective agents, and their anti­oxidant and enzyme inhibitory activities (Lin *et al.*, 2002 ▸; Bhat *et al.*, 2005 ▸; Trivedi *et al.*, 2007 ▸; Lahtchev *et al.*, 2008 ▸; Aneja *et al.*, 2018 ▸).

Chalcone as a privileged structure in medicinal chemistry has been reviewed by Zhuang *et al.* (2017 ▸). A comprehensive review of chalcone derivatives as anti­leishmanial agents has also been published (de Mello *et al.*, 2018 ▸). The crystal structures of (2*E*)-1-(4-meth­ylphen­yl)-3-(4-nitro­phen­yl)prop-2-en-1-one (Butcher *et al.*, 2007 ▸), (2*E*)-1-(3-bromo­phen­yl)-3-(4,5-dimeth­oxy-2-nitro­phen­yl)prop-2-en-1-one (Jasinski *et al.*, 2010 ▸), (2*E*)-3-(3-nitro­phen­yl)-1-[4-(piperidin-1-yl)phen­yl]prop-2-en-1-one (Fun *et al.*, 2012 ▸) and 4′-di­meth­ylamino-3-nitro­chalcone, 3-di­meth­ylamino-3′-nitro­chalcone and 3′-nitro­chalcone (Hall *et al.*, 2020 ▸) have been reported.

**Figure 1:**
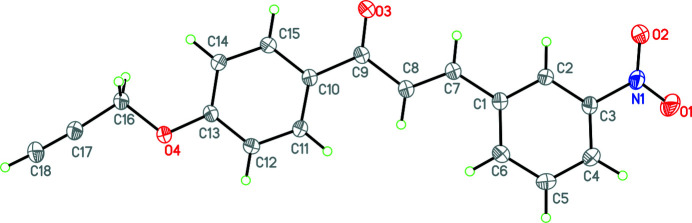
Diagram of mol­ecules showing the atom-labelling scheme. Atomic displacement parameters are at the 30% probability level.

The present work describes the synthesis and crystal structure of the title compound 3-(3-nitro­phen­yl)-1-[4-(prop-2-yn­yloxy)phen­yl]prop-2-en-1-one (Fig. 1 ▸), which crystallizes in the triclinic space group *P* 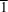 with one mol­ecule in the asymmetric unit. It consists both a 3-nitro­phenyl group and a (prop-2-yn-1-yl­oxy)benzene group linked to a central chalcone moiety. Even though the C—N bond length is 1.4706 (17) Å and thus single, the nitro group is almost coplanar with its phenyl ring [dihedral angle of 18.94 (6)°] as a result of the steric clash between O1 and H4 and between O2 and H2, respectively. The chalcone group is planar (average deviation from plane of 0.004 Å) and makes dihedral angles of 7.69 (8) and 10.96 (6)° with the 3-nitro­phenyl ring and the phenyl ring of the (prop-2-yn-1-yl­oxy)benzene group, respectively. Lastly, the twist between the two phenyl rings which are linked by the chalcone is 19.22 (5)°.

**Figure 2:**
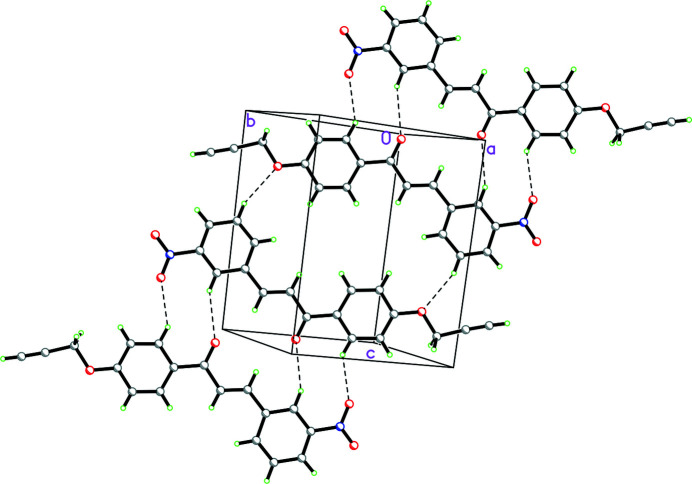
Packing diagram for the title compound showing the C–H⋯O inter­actions linking the mol­ecules into sheets in the (21 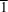 ) plane.

The mol­ecules are linked by C–H⋯O inter­actions (Table 1 ▸), which form sheets in the (21 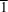 ) plane as shown in Fig. 2 ▸ *.* There are no π–π inter­actions between the phenyl rings.

## Synthesis and crystallization

**Figure 3:**
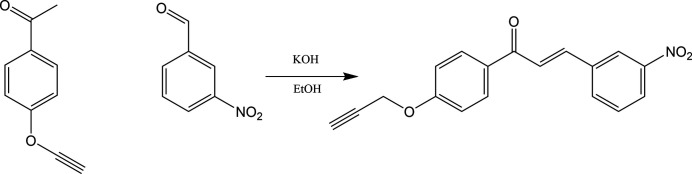
Reaction scheme for the synthesis of the title compound.

A well-stirred solution of 1-[4-(prop-2-yn­yloxy)phen­yl]ethan­one (1 g, 1 mmol) in 20 ml of ethanol was added slowly to alcoholic potassium hydroxide (0.48 g, 1.5 mmol). To this solution, *m*-nitro benzaldehyde (1.03 g, 1.2 mmol) was added. The resulting mixture was stirred at room temperature for 30 min. Then, the separated solid from the reaction mixture was filtered, washed with cold water, dried and recrystallized from ethanol:di­methyl­formamide mixture (9:1). Golden yellow crystals (yield: 86%, m.p. 453–454 K). The reaction scheme is shown in Fig. 3 ▸. FT–IR: νmax, cm^−1^ (KBr): 2987 (C—H aliphatic), 2117 (C≡C str), 1650 (C=O), 1518 (asym NO_2_ stretch),1444 (sym NO_2_ stretch), 1252 (C—O stretch); ^1^H NMR (400 MHz, CDCl3, δ p.p.m.): 7.55 (*d*, 1H, *J* = 15.7 Hz, olefinic-β), 7.36 (*d*, 2*H*, *J* = 8.8 Hz, Ar—H), 7.28 (*d*, 2H, *J* = 8.6 Hz, Ar—H), 7.16 (*d*, 2H, *J* = 8.8 Hz, Ar—H), 7.09 (*d*, 2H, *J* = 8.3 Hz, Ar—H), 6.73 (*d*, 1H, *J* = 15.7 Hz, olefinic-α), 4.46 (*s*, 2H, O—CH_2_), 2.79 (*s*, 1H, acetyl­ene proton).

## Refinement

Crystal data, data collection and structure refinement details for the title compound are summarized in Table 2 ▸.

## Supplementary Material

Crystal structure: contains datablock(s) I. DOI: 10.1107/S2414314622009579/bt4126sup1.cif

Structure factors: contains datablock(s) I. DOI: 10.1107/S2414314622009579/bt4126Isup2.hkl

Supporting information file. DOI: 10.1107/S2414314622009579/bt4126Isup3.cml

CCDC reference: 2210152

Additional supporting information:  crystallographic information; 3D view; checkCIF report

## Figures and Tables

**Table 1:** Hydrogen-bond geometry (Å, °)

*D*—H⋯*A*	*D*—H	H⋯*A*	*D*⋯*A*	*D*—H⋯*A*
C18—H18*A*⋯O2^i^	0.901 (18)	2.519 (18)	3.3927 (18)	163.6 (15)

Symmetry code: (i) 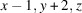 .

**Table 2:** Experimental details

Crystal data	
Chemical formula	C_18_H_13_NO_4_
*M*_r_	307.29
Crystal system, space group	Triclinic, *P* 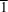
Temperature (K)	100
*a*, *b*, *c* (Å)	7.6534 (16), 8.6079 (15), 11.369 (2)
α, β, γ (°)	94.433 (7), 97.953 (8), 97.019 (7)
*V* (Å^3^)	732.8 (3)
*Z*	2
Radiation type	Mo *K*α
μ (mm^−1^)	0.10
Crystal size (mm)	0.33 × 0.19 × 0.14

Data collection	
Diffractometer	Bruker APEXII CCD
Absorption correction	Multi-scan (*SADABS*; Bruker, 2016 ▸)
*T*_min_, *T*_max_	0.634, 0.729
No. of measured, independent and observed [*I* > 2σ(*I*)] reflections	47166, 3638, 2700
*R*_int_	0.089
(sin θ/λ)_max_ (Å^−1^)	0.667

Refinement	
*R*[*F* ^2^ > 2σ(*F* ^2^)], *wR*(*F* ^2^), *S*	0.046, 0.137, 1.08
No. of reflections	3638
No. of parameters	212
H-atom treatment	H atoms treated by a mixture of independent and constrained refinement
Δρ_max_, Δρ_min_ (e Å^−3^)	0.28, −0.21

Computer programs: *APEX2* and *SAINT* (Bruker, 2016 ▸), *SHELXT* (Sheldrick, 2015*a* ▸), *SHELXL2018/3* (Sheldrick, 2015*b* ▸) and *SHELXTL* (Sheldrick, 2008 ▸).

## full crystallographic data

### Crystal data

**Table:**

C_18_H_13_NO_4_	*Z* = 2
*M**_r_* = 307.29	*F*(000) = 320
Triclinic, *P*1	*D*_x_ = 1.393 Mg m^−^^3^
*a* = 7.6534 (16) Å	Mo *K*α radiation, λ = 0.71073 Å
*b* = 8.6079 (15) Å	Cell parameters from 9976 reflections
*c* = 11.369 (2) Å	θ = 2.7–32.9°
α = 94.433 (7)°	µ = 0.10 mm^−^^1^
β = 97.953 (8)°	*T* = 100 K
γ = 97.019 (7)°	Prism, yellow
*V* = 732.8 (3) Å^3^	0.33 × 0.19 × 0.14 mm

### Data collection

**Table:**

Bruker APEXII CCD diffractometer	2700 reflections with *I* > 2σ(*I*)
φ and ω scans	*R*_int_ = 0.089
Absorption correction: multi-scan (SADABS; Bruker, 2016)	θ_max_ = 28.3°, θ_min_ = 2.4°
*T*_min_ = 0.634, *T*_max_ = 0.729	*h* = −10→10
47166 measured reflections	*k* = −11→10
3638 independent reflections	*l* = −15→15

### Refinement

**Table:**

Refinement on *F*^2^	Primary atom site location: dual
Least-squares matrix: full	Secondary atom site location: difference Fourier map
*R*[*F*^2^ > 2σ(*F*^2^)] = 0.046	Hydrogen site location: mixed
*wR*(*F*^2^) = 0.137	H atoms treated by a mixture of independent and constrained refinement
*S* = 1.08	*w* = 1/[σ^2^(*F*_o_^2^) + (0.0642*P*)^2^ + 0.1082*P*] where *P* = (*F*_o_^2^ + 2*F*_c_^2^)/3
3638 reflections	(Δ/σ)_max_ < 0.001
212 parameters	Δρ_max_ = 0.28 e Å^−^^3^
0 restraints	Δρ_min_ = −0.21 e Å^−^^3^

### Special details

**Table:**

Geometry. All esds (except the esd in the dihedral angle between two l.s. planes) are estimated using the full covariance matrix. The cell esds are taken into account individually in the estimation of esds in distances, angles and torsion angles; correlations between esds in cell parameters are only used when they are defined by crystal symmetry. An approximate (isotropic) treatment of cell esds is used for estimating esds involving l.s. planes.
Refinement. The acetylenic H atom was freely refined. All remaining hydrogen atoms were placed geometrically and refined as riding atoms with their *U*_iso_ values 1.2 times that of their attached atoms.

### Fractional atomic coordinates and isotropic or equivalent isotropic displacement parameters (Å^2^)

**Table:**

	*x*	*y*	*z*	*U*_iso_*/*U*_eq_	
O1	0.8224 (2)	−0.53179 (13)	0.40582 (11)	0.0732 (4)	
O2	0.78869 (16)	−0.48667 (12)	0.22192 (10)	0.0557 (3)	
O3	0.38666 (16)	0.19330 (12)	0.03294 (9)	0.0557 (3)	
O4	0.12863 (14)	0.82184 (10)	0.22678 (8)	0.0419 (3)	
N1	0.79204 (16)	−0.44535 (13)	0.32710 (11)	0.0436 (3)	
C1	0.63484 (18)	−0.05300 (15)	0.30785 (12)	0.0358 (3)	
C2	0.67213 (17)	−0.20372 (14)	0.27518 (11)	0.0342 (3)	
H2A	0.639784	−0.249615	0.195197	0.041*	
C3	0.75705 (18)	−0.28509 (15)	0.36138 (12)	0.0362 (3)	
C4	0.8101 (2)	−0.22355 (17)	0.47820 (12)	0.0446 (3)	
H4A	0.868961	−0.282414	0.535011	0.054*	
C5	0.7751 (2)	−0.07331 (18)	0.51018 (13)	0.0518 (4)	
H5A	0.811026	−0.027252	0.589874	0.062*	
C6	0.6882 (2)	0.00972 (17)	0.42650 (13)	0.0469 (4)	
H6A	0.663892	0.112233	0.450078	0.056*	
C7	0.54529 (18)	0.03375 (15)	0.21647 (12)	0.0378 (3)	
H7A	0.524932	−0.013883	0.136707	0.045*	
C8	0.49007 (19)	0.17290 (15)	0.23512 (12)	0.0402 (3)	
H8A	0.505167	0.222444	0.314155	0.048*	
C9	0.40553 (19)	0.25253 (15)	0.13558 (12)	0.0386 (3)	
C10	0.34071 (18)	0.40617 (14)	0.16303 (11)	0.0345 (3)	
C11	0.32576 (18)	0.46816 (15)	0.27815 (11)	0.0366 (3)	
H11A	0.365827	0.414762	0.344742	0.044*	
C12	0.25357 (19)	0.60589 (15)	0.29624 (11)	0.0382 (3)	
H12A	0.242477	0.645757	0.374749	0.046*	
C13	0.19719 (17)	0.68610 (14)	0.19960 (11)	0.0343 (3)	
C14	0.2132 (2)	0.62771 (16)	0.08447 (12)	0.0418 (3)	
H14A	0.176033	0.682647	0.018101	0.050*	
C15	0.2840 (2)	0.48861 (16)	0.06802 (12)	0.0418 (3)	
H15A	0.293985	0.448410	−0.010617	0.050*	
C16	0.0652 (2)	0.90385 (15)	0.12751 (12)	0.0412 (3)	
H16A	−0.037244	0.838377	0.076985	0.049*	
H16B	0.160640	0.926181	0.078194	0.049*	
C17	0.01114 (18)	1.05106 (15)	0.17380 (12)	0.0397 (3)	
C18	−0.0334 (2)	1.17155 (17)	0.20482 (14)	0.0466 (4)	
H18A	−0.067 (2)	1.266 (2)	0.2248 (15)	0.057 (5)*	

### Atomic displacement parameters (Å^2^)

**Table:**

	*U*^11^	*U*^22^	*U*^33^	*U*^12^	*U*^13^	*U*^23^
O1	0.1189 (11)	0.0461 (6)	0.0560 (7)	0.0324 (7)	−0.0072 (7)	0.0173 (5)
O2	0.0791 (8)	0.0448 (6)	0.0449 (6)	0.0280 (5)	0.0012 (5)	−0.0014 (5)
O3	0.0887 (9)	0.0452 (6)	0.0366 (6)	0.0323 (6)	0.0041 (5)	−0.0009 (4)
O4	0.0605 (6)	0.0339 (5)	0.0339 (5)	0.0210 (4)	0.0040 (4)	0.0022 (4)
N1	0.0506 (7)	0.0363 (6)	0.0440 (7)	0.0146 (5)	−0.0018 (5)	0.0069 (5)
C1	0.0395 (7)	0.0333 (6)	0.0357 (7)	0.0103 (5)	0.0049 (5)	0.0040 (5)
C2	0.0384 (7)	0.0328 (6)	0.0316 (6)	0.0087 (5)	0.0027 (5)	0.0026 (5)
C3	0.0397 (7)	0.0333 (6)	0.0372 (7)	0.0102 (5)	0.0052 (5)	0.0063 (5)
C4	0.0535 (8)	0.0465 (8)	0.0352 (7)	0.0153 (6)	0.0012 (6)	0.0090 (6)
C5	0.0691 (10)	0.0530 (9)	0.0320 (7)	0.0183 (7)	−0.0018 (7)	−0.0026 (6)
C6	0.0633 (9)	0.0391 (7)	0.0388 (7)	0.0179 (7)	0.0032 (6)	−0.0021 (6)
C7	0.0457 (7)	0.0333 (6)	0.0353 (7)	0.0121 (5)	0.0040 (5)	0.0016 (5)
C8	0.0514 (8)	0.0340 (7)	0.0359 (7)	0.0148 (6)	0.0025 (6)	0.0014 (5)
C9	0.0484 (8)	0.0324 (6)	0.0366 (7)	0.0130 (5)	0.0061 (6)	0.0021 (5)
C10	0.0406 (7)	0.0296 (6)	0.0339 (6)	0.0096 (5)	0.0038 (5)	0.0025 (5)
C11	0.0472 (7)	0.0324 (6)	0.0317 (6)	0.0115 (5)	0.0037 (5)	0.0066 (5)
C12	0.0514 (8)	0.0343 (6)	0.0299 (6)	0.0109 (6)	0.0064 (5)	0.0014 (5)
C13	0.0396 (7)	0.0281 (6)	0.0352 (6)	0.0090 (5)	0.0033 (5)	0.0007 (5)
C14	0.0596 (9)	0.0371 (7)	0.0307 (6)	0.0188 (6)	0.0018 (6)	0.0047 (5)
C15	0.0603 (9)	0.0365 (7)	0.0305 (6)	0.0177 (6)	0.0044 (6)	0.0011 (5)
C16	0.0526 (8)	0.0343 (7)	0.0368 (7)	0.0160 (6)	−0.0016 (6)	0.0028 (5)
C17	0.0429 (7)	0.0361 (7)	0.0408 (7)	0.0107 (6)	0.0019 (6)	0.0062 (5)
C18	0.0518 (9)	0.0378 (7)	0.0527 (9)	0.0170 (6)	0.0063 (7)	0.0052 (6)

### Geometric parameters (Å, º)

**Table:**

O1—N1	1.2232 (15)	C8—C9	1.4815 (18)
O2—N1	1.2168 (16)	C8—H8A	0.9500
O3—C9	1.2189 (16)	C9—C10	1.4940 (17)
O4—C13	1.3693 (14)	C10—C15	1.3866 (17)
O4—C16	1.4362 (15)	C10—C11	1.3997 (18)
N1—C3	1.4706 (17)	C11—C12	1.3809 (17)
C1—C2	1.3957 (17)	C11—H11A	0.9500
C1—C6	1.3996 (19)	C12—C13	1.3888 (17)
C1—C7	1.4680 (18)	C12—H12A	0.9500
C2—C3	1.3838 (17)	C13—C14	1.3924 (18)
C2—H2A	0.9500	C14—C15	1.3835 (18)
C3—C4	1.3780 (19)	C14—H14A	0.9500
C4—C5	1.384 (2)	C15—H15A	0.9500
C4—H4A	0.9500	C16—C17	1.4627 (18)
C5—C6	1.380 (2)	C16—H16A	0.9900
C5—H5A	0.9500	C16—H16B	0.9900
C6—H6A	0.9500	C17—C18	1.1746 (19)
C7—C8	1.3295 (17)	C18—H18A	0.901 (18)
C7—H7A	0.9500		
			
C13—O4—C16	116.23 (10)	O3—C9—C10	120.22 (12)
O2—N1—O1	122.80 (12)	C8—C9—C10	118.90 (11)
O2—N1—C3	118.79 (11)	C15—C10—C11	118.06 (11)
O1—N1—C3	118.40 (12)	C15—C10—C9	117.85 (11)
C2—C1—C6	118.12 (12)	C11—C10—C9	124.01 (11)
C2—C1—C7	118.90 (11)	C12—C11—C10	120.89 (11)
C6—C1—C7	122.97 (12)	C12—C11—H11A	119.6
C3—C2—C1	118.80 (12)	C10—C11—H11A	119.6
C3—C2—H2A	120.6	C11—C12—C13	119.98 (12)
C1—C2—H2A	120.6	C11—C12—H12A	120.0
C4—C3—C2	123.22 (12)	C13—C12—H12A	120.0
C4—C3—N1	118.22 (11)	O4—C13—C12	115.58 (11)
C2—C3—N1	118.56 (11)	O4—C13—C14	124.35 (11)
C3—C4—C5	117.94 (12)	C12—C13—C14	120.07 (11)
C3—C4—H4A	121.0	C15—C14—C13	119.11 (12)
C5—C4—H4A	121.0	C15—C14—H14A	120.4
C6—C5—C4	120.11 (13)	C13—C14—H14A	120.4
C6—C5—H5A	119.9	C14—C15—C10	121.89 (12)
C4—C5—H5A	119.9	C14—C15—H15A	119.1
C5—C6—C1	121.80 (13)	C10—C15—H15A	119.1
C5—C6—H6A	119.1	O4—C16—C17	108.45 (11)
C1—C6—H6A	119.1	O4—C16—H16A	110.0
C8—C7—C1	125.96 (12)	C17—C16—H16A	110.0
C8—C7—H7A	117.0	O4—C16—H16B	110.0
C1—C7—H7A	117.0	C17—C16—H16B	110.0
C7—C8—C9	121.55 (12)	H16A—C16—H16B	108.4
C7—C8—H8A	119.2	C18—C17—C16	176.40 (14)
C9—C8—H8A	119.2	C17—C18—H18A	177.0 (11)
O3—C9—C8	120.87 (12)		
			
C6—C1—C2—C3	1.0 (2)	C7—C8—C9—C10	177.91 (13)
C7—C1—C2—C3	179.80 (12)	O3—C9—C10—C15	−9.4 (2)
C1—C2—C3—C4	−1.3 (2)	C8—C9—C10—C15	171.57 (13)
C1—C2—C3—N1	178.51 (11)	O3—C9—C10—C11	167.42 (14)
O2—N1—C3—C4	−161.56 (14)	C8—C9—C10—C11	−11.6 (2)
O1—N1—C3—C4	18.8 (2)	C15—C10—C11—C12	1.2 (2)
O2—N1—C3—C2	18.64 (19)	C9—C10—C11—C12	−175.62 (13)
O1—N1—C3—C2	−160.96 (13)	C10—C11—C12—C13	−1.0 (2)
C2—C3—C4—C5	0.5 (2)	C16—O4—C13—C12	−178.35 (11)
N1—C3—C4—C5	−179.33 (13)	C16—O4—C13—C14	2.0 (2)
C3—C4—C5—C6	0.6 (2)	C11—C12—C13—O4	−179.60 (12)
C4—C5—C6—C1	−0.7 (3)	C11—C12—C13—C14	0.1 (2)
C2—C1—C6—C5	−0.1 (2)	O4—C13—C14—C15	−179.70 (13)
C7—C1—C6—C5	−178.78 (15)	C12—C13—C14—C15	0.7 (2)
C2—C1—C7—C8	175.05 (13)	C13—C14—C15—C10	−0.5 (2)
C6—C1—C7—C8	−6.3 (2)	C11—C10—C15—C14	−0.4 (2)
C1—C7—C8—C9	178.18 (13)	C9—C10—C15—C14	176.58 (13)
C7—C8—C9—O3	−1.1 (2)	C13—O4—C16—C17	−175.40 (11)

### Hydrogen-bond geometry (Å, º)

**Table:**

*D*—H···*A*	*D*—H	H···*A*	*D*···*A*	*D*—H···*A*
C18—H18*A*···O2^i^	0.901 (18)	2.519 (18)	3.3927 (18)	163.6 (15)

Symmetry code: (i) *x*−1, *y*+2, *z*.
